# Effect of Smut Infection on the Photosynthetic Physiological Characteristics and Related Defense Enzymes of Sugarcane

**DOI:** 10.3390/life12081201

**Published:** 2022-08-08

**Authors:** Xiupeng Song, Fenglian Mo, Meixin Yan, Xiaoqiu Zhang, Baoqing Zhang, Xing Huang, Dongmei Huang, Yangfei Pan, Krishan K. Verma, Yang-Rui Li

**Affiliations:** 1 Sugarcane Research Institute, Guangxi Academy of Agricultural Sciences/Key Laboratory of Sugarcane Biotechnology and Genetic Improvement (Guangxi), Ministry of Agriculture and Rural Affairs/Guangxi Key Laboratory of Sugarcane Genetic Improvement, Nanning 530007, China; 2 College of Agriculture, Guangxi University, Nanning 530004, China

**Keywords:** chlorophyll fluorescence efficiency, photosynthetic responses, enzyme activity, sugarcane, smut

## Abstract

Pathogen infection seriously affects plant development and crop productivity, sometimes causing total crop failure. In this study, artificial stab inoculation was used to inoculate sugarcane smut. The changes in leaf gas exchange, chlorophyll fluorescence variables, and related defense enzyme activities were measured in sugarcane cultivar ROC22 after pathogen infection. The results showed that the net photosynthetic rate (Pn), stomatal conductance (gs), and transpiration rate (Tr) downregulated in the first three days after smut infection and upregulated on the fourth day; intercellular CO_2_ concentration (Ci) increased in the first three days of smut infection and reduced on the fourth day. The chlorophyll fluorescence parameters, i.e., Fo, Fm, Fv/Fm, Fs, and Fv′/Fm′ decreased at the initial stage of pathogen infection but increased rapidly up to 3 days after smut infection. It can be seen that sugarcane seedlings showed a positive response to pathogen infection. The correlation coefficient relationship between Pn, gs, and Tr reached above 0.800, showing a significant correlation; Ci was positively correlated with Fv′/Fm′ and ΦPSII, reaching above 0.800 and showing a significant correlation; Fo positively correlated with Fv/Fm, Fs, and ETR; Fv /Fm was positively correlated with Fv′/Fm′; Fs significantly correlated with Fv′/Fm′; and Fv′/Fm′ positively correlated with ΦPSII. After inoculation with smut, the related defense enzymes, i.e., POD, SOD, PPO, and PAL, were increased and upregulated; photosynthetic parameters can be associated with an increase in enzymatic activities. The results of this study will help to further study of the response mechanism to smut in the sugarcane growing period and provide a theoretical reference for sugarcane resistance to smut breeding.

## 1. Introduction

Sugarcane is an important bioenergy crop grown worldwide. Its sugar production accounts for 80% of global sugar production and 92% of China’s total sugar production [1,2]. Disease is one of the main factors causing the loss of sugarcane yield and sugar content [3]. A variety of pathogens can infect sugarcane during the growth process. Pathogens can accumulate in the sugarcane germplasm, leading to variability, uneven growth, reduced stem weight, sugar loss, and so on. Sugarcane in different countries has about 150 diseases, of which, smut is the main disease and can cause a 20–50% loss of sugarcane production [4,5]. The most apparent symptom of sugarcane infection in the late stage of smut is the extraction of smut whip at the tail of the cane, but there is no obvious morphological feature in the early stage of infection. Studies have shown that pathogen infection can seriously affect the photosynthetic physiological responses in crops [5,6]. There are various studies on the changes in physiological traits of sugarcane after smut infection [7,8], but there are no reports of the photosynthetic responses of sugarcane seedlings in the early stage of smut infection.

When pathogens infect plants, plant cells produce a series of physiological and biochemical changes to prevent the infection. These changes include early defense reactions after plant susceptibility such as thickening of the corpus callosum, changes in protective enzyme activities, induction and accumulation of disease-related proteins, and hormonal and metabolic disorders [5,9,10,11]. In these defense reactions, enzymatic activities are most active and closely related to plant disease resistance. Under normal physiological conditions, various enzymes in plants are generally in a dynamic equilibrium state. When pathogens infect plant cells, a specific type of enzyme changes the action of the mechanisms, thereby losing its original equilibrium state and harming the organism [5]. Therefore, it is of great significance to study the resistance of the host to the disease when a particular disease infects it. The enzymes involved in disease resistance include peroxidase (POD), superoxide dismutase (SOD), polyphenol oxidase (PPO), and phenylalanine ammonia-lyase (PAL) [5,12]. After studying the relationship between various physiological and biochemical metabolic reactions of the host and plant disease resistance, it is concluded that SOD, POD, and other related enzymes can resist the damage of reactive oxygen and oxygen free radicals to the cell membrane system, while PAL and PPO can promote the production of various secondary metabolites in plants, thereby preventing the invasion and reproduction of pathogens [13,14].

In the present study, after inoculation with smut pathogen, changes in parameters between sugarcane plant leaves, such as the intercellular CO_2_ concentration (Ci), net photosynthetic rate (Pn), transpiration rate (Tr), stomatal conductance (gs), initial fluorescence (Fo), the maximum potential quantum efficiency of photosystem II (Fv/Fm), minimum fluorescence under light (Fo′), non-photochemical quenching coefficient (qNP), maximum light energy conversion efficiency (Fv′/Fm′), steady-state fluorescence (Fs), electron transport rate (ETR), photochemical quenching coefficient (qP), and maximum fluorescence under light (Fm′), were measured. The preliminary analysis of relationships with resistance and the detection of POD, SOD, PPO, and PAL activities after smut infection, the effects of smut infection on photosynthetic physiological changes, and resistance-related enzyme activities and response mechanisms were explored, providing a theoretical basis for further research on the resistance mechanism and disease management of sugarcane to smut.

## 2. Materials and Methods

### 2.1. Plant Materials, Growth Conditions, and Treatments

The sugarcane cultivar ROC22 was used in this experiment, the most prevalent variety in China. The experiment was completed in the greenhouse of Guangxi University, Nanning, Guangxi, China. The healthy single bud of the test material was disinfected with hot water treatment and used for sowing in the sand. After the emergence of the seedlings, the healthy seedlings with strong growth and consistency were strictly selected and planted in plastic barrels (40 cm upper diameter, 30 cm lower diameter, and 40 cm height). The controlled moisture content was 70% of the maximum water holding capacity in the field, and the experiment was carried out when the seedlings had 6–7 true leaves. The smut pathogen teliospores were collected from the sugarcane base of the Agricultural College of Guangxi University, heated at 40 °C (1 h), stored in sterilized paper bags, and stored at 4 °C for further use.

The test was carried out by the stab inoculation method, and the pathogen teliospores germination rate was more than 95%. They were collected and diluted with sterile water into a spore suspension with a concentration of 5 × 10^6^ spores/mL using a sterile syringe. The sterile syringe was stabbed four times into the sugarcane at the growing stage, and then a 4-drop suspension (about 50 μL) was added along the leaf sheath. The control group replaced the spore suspension with sterilized ddH_2_O. Inoculated once every day, the photosynthetic physiological indexes of the sugarcane (+1) leaves inoculated for 1, 2, 3, and 4 days were uniformly measured up to the 4th day after inoculation. The enzyme activity was measured 1, 3, 5, 7, and 9 days after smut inoculation.

### 2.2. Measurement of Leaf Gas Exchange and Chlorophyll Fluorescence

Photosynthetic responses were measured from sugarcane cultivar ROC 22 1, 2, 3, and 4 days after smut inoculation. The photosynthetic rate (Pn), stomatal conductance (gs), transpiration rate (Tr), and internal CO_2_ concentration (Ci) were observed using a portable photosynthesis system (Li-6400xt, LICOR, Lincoln, NE, USA) from a photosynthetically fully mature leaf (+1). For each treatment and control, a minimum of five (*n* = 5) measurements were recorded between 10:00–11:00 am. The photosynthetic photon flux density ((PPFD) 1000 µmol m^−2^s^−1^), the leaf chamber temperature (35 °C), and the flow rate (500 µmol s^−1^) were used while recording photosynthetic leaf gas exchange.

Chlorophyll fluorescence was measured by using an FMS-2 Modulate fluorometer (Hansatech, UK). Dark-adapted leaf (30 min), initial fluorescence (Fo) with weak measurement light, maximum fluorescence (Fm) with saturated pulsed light (9000 μmol m^−2^s^−1^), and photochemical light (1200 μmol m^−2^s^−1^) were used to determine the steady-state fluorescence (Fs), maximum (Fm′), and minimum fluorescence (Fo′) under the light. The PSII maximum variable fluorescence (Fv = Fm − Fo), maximum variable fluorescence under light (Fv′ = Fm − Fo′), maximum light energy conversion efficiency (Fv/Fm), actual light energy conversion efficiency (ΦPSII = (Fm′ − Fs)/Fm′), maximum light energy conversion efficiency (Fv′/Fm′) of the PSII reaction center under light adaptation, electron transport rate (ETR), non-photochemical quenching coefficient (qNP), and photochemical quenching coefficient (qP) were calculated by the chlorophyll fluorescence parameters. Each treatment was replicated thrice (*n* = 3).

### 2.3. Enzyme Extraction

For the determination of antioxidative enzyme activities, 1 g of leaf samples were homogenized in 5 mL of pre-cooled sodium borate buffer (pH 8.8) containing 1 mM EDTA, 5 mM β-mercaptoethanol, and 4% (*w*/*v*) PVP, incubated at 4 °C for 5 min. After incubation, the homogenate was centrifuged (12,000× *g*) for 20 min at 4 °C, and the supernatant was used for the subsequent estimation of POD, SOD, PPO, and PAL activities. The enzyme activities were expressed as U g^−1^ FW. 

#### 2.3.1. Determination of Peroxidase and Superoxide Dismutase Activity

The peroxidase (POD) activity was assessed according to Nakano and Asada [15] with slight modifications. The reaction mixture contained 0.1M phosphate buffer (pH 5.8) and 18 mM guaiacol, mixed with the 50 µL of enzyme extract followed by the addition of 2.5% H_2_O_2_ (*v*/*v*). The absorbance of the mixture was measured at 470 nm by a UV-spectrophotometer. The specific POD activity was calculated by using the below formula and expressed as U g^−1^ FW.

POD activity (U g^−1^ FW) = (∆A_470_ × Vt/(W × Vs × 0.01 × t)

where ∆A_470_ indicates the time for the change in absorbance, Vt is the total volume of the reaction mixture, W is the sample fresh weight, Vs is the volume of the crude enzyme extract, and t is the reaction time (min).

Superoxide dismutase (SOD) activity was determined in terms of its capacity for 50% inhibition of the photochemical reduction in NBT monitored at 560 nm as previously described by Giannopolitis and Reis [16]. The reaction mixture contained 50 mM phosphate buffer (pH 7.8), 13 mM methionine, 63 µM NBT, 1.3 µM riboflavin, and 0.1 mM EDTA mixed with 0.5 mL of the enzyme solution. The specific SOD activity was calculated by using the below formula and expressed as U g^−1^ FW.

SOD activity (U g^−1^ FW) = (∆A_560_ × Vt/(W × Vs × 0.05 × t)

where ∆A_560_ is the change in absorbance, Vt is the total volume of the reaction mixture, W is the fresh weight of the sample, Vs is the volume of the crude enzyme, and t is the reaction time (min).

#### 2.3.2. Quantification of Polyphenol Oxidase and Phenylalanine Ammonia-Lyase Activity

Polyphenol oxidase (PPO) activity was assayed as described by Zhang and Shao [17] with minor modifications using a mixture (5 mL) containing 0.1 M sodium phosphate buffer (pH 6.8), 0.02 M catechol, and crude enzyme extract. The enzyme extract was added to start the reaction. A heat-killed crude enzyme was used in the control. The absorbance at 420 nm was observed for 3 min at 30-s intervals and the values per minute were calculated. The results were presented as U g^−1^ FW.

Phenylalanine ammonia-lyase (PAL) was quantified by the procedure described by Aoki et al. [18]. The reaction mixture (3 mL) consisted of 0.02 M L-phenylalanine (0.75 mL), 0.01 M borate buffer (2.15 mL, pH 8.8) and 0.1 mL of crude enzyme extract. Phenylalanine conversion into cinnamic acid was estimated at 290 nm and expressed as U g^−1^ FW. The samples were incubated at 30 °C for 1 h. In the control, the enzyme extract was replaced with 1 mL of borate buffer. The reaction was stopped in an icebox. One activity unit was defined as a change in absorbance of 0.01 at 290 nm. 

### 2.4. Data Processing

The analytical data were analyzed using Microsoft Excel and SPSS 15.0 software, and the significance test was performed using Duncan’s new complex range method.

## 3. Results

### 3.1. Effect of Smut Infection on Photosynthetic Leaf Gas Exchange 

With the prolongation of the infection time of the smut pathogen, the photosynthesis rate (Pn) of sugarcane seedlings showed a change of “rise-lower-rise”, which was significantly decreased when compared with the control on the third day; it showed a substantial increase on the fourth day after smut inoculation. It can be seen that the smut infection attacked the normal photosynthesis of sugarcane seedlings and promoted photosynthetic improvement. This may be the vital reason why it affects the early growth of sugarcane seedlings (Table 1). The changing trend of stomatal conductance (gs) of sugarcane seedlings is consistent with the changing trend of Pn, and there is a positive correlation between Pn and gs. However, compared with the control, the changes in the gs of sugarcane seedlings after pathogen infection were not apparent. It can be seen that stomatal factors do not entirely control the changes in the Pn rate (Table 1).

The intracellular CO_2_ concentration (Ci) after smut infection showed a trend of increasing first and then reducing, as compared to control plants (Table 1). The enhancement was the largest after the third day of smut inoculation, significantly different from the control condition. This situation occurred when the difference in gs was not noticeable, and the Pn was decreased. It is speculated that the photorespiration of sugarcane seedlings is enhanced after smut infestation, which causes an increase in the Ci level.

The transpiration rate (Tr) of plants reflects the degree of water loss in the aboveground part, which can be used to observe the ability of plants to regulate water. This index is closely associated with multiple physiological and metabolic pathways and also indirectly reflects the regulation of the plant’s photosynthesis. It can be seen from Table 1 that with the prolongation of infection time, the Tr of sugarcane leaves decreased and then increased. The treatment and control trends were consistent, but the difference between the treatment and control did not increase significantly (Table 1).

Photosynthetic water use efficiency (WUE), the amount of CO_2_ fixed by plant consumption per unit of weight water, usually uses Pn/Tr to indicate the level of plant water-use efficiency. When the water supply to the plant is low, the plant generally tends to achieve a higher WUE by adjusting the openness of the stomata while maintaining high Pn; when the environmental water supply is especially insufficient, the plant can reduce the transpiration rate to improve WUE. It can be seen from Table 1 that the smut infection reduced the WUE of sugarcane plants, and the downregulation was most significant on the third day after smut inoculation.

### 3.2. Effect of Smut Infection on Chlorophyll Fluorescence Variables

**Figure 1:**
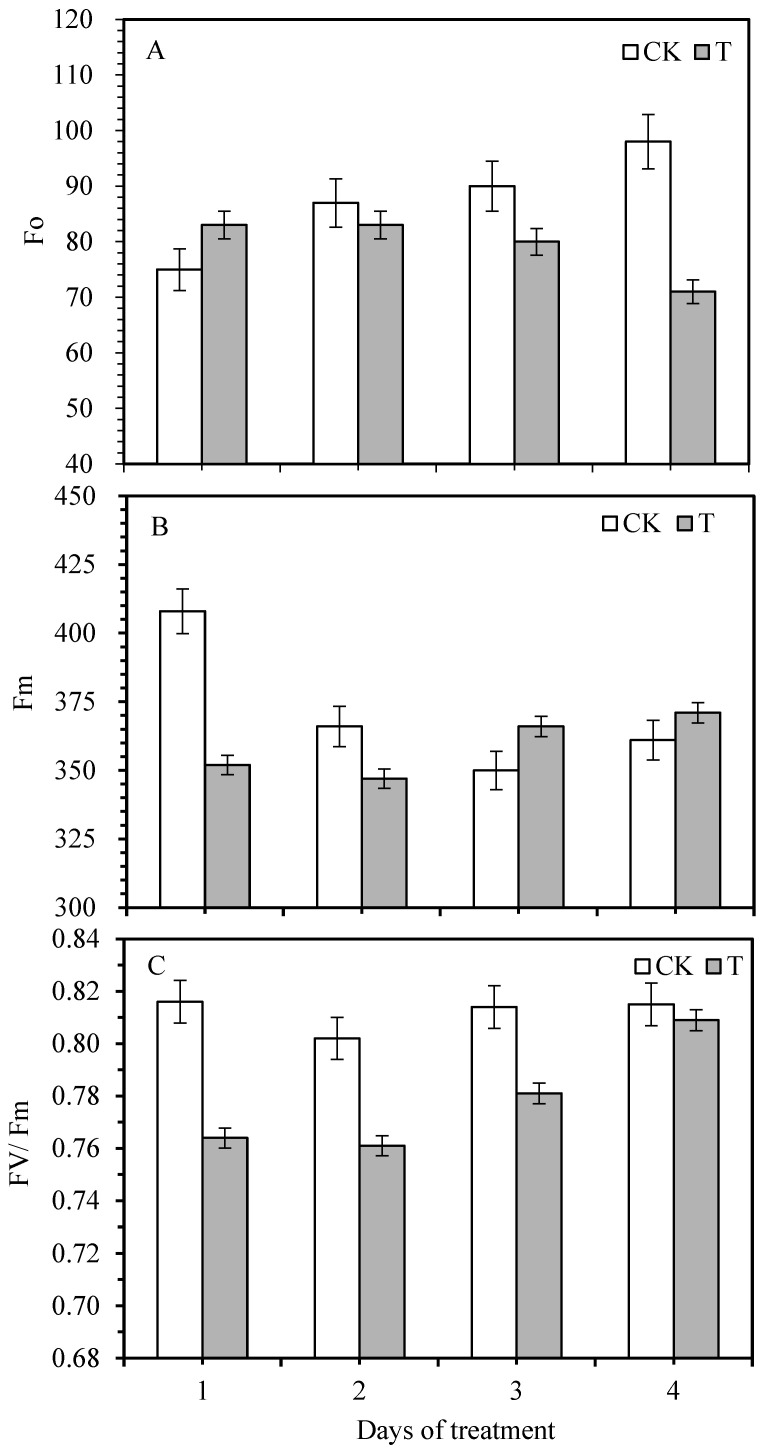
The variation of minimum ((**A**), Fo), maximum ((**B**), Fm), and optimum chlorophyll fluorescence yield of PS II ((**C**), Fv/Fm) of sugarcane cv. ROC22 plant leaves in response to smut inoculation at different time periods. CK—control, T—smut inoculation.

Minimal chlorophyll fluorescence (Fo) is the fluorescence yield of the photosystem II (PS II) reaction center when it is completely open. The changes are closely interconnected to the leaf chlorophyll concentration, showing the permanent damage of stress to PSII in the plant leaves. The maximum fluorescence yield (Fm) reflects the electron transfer through the PSII, which is the fluorescence yield when the PSII reaction center is completely closed. The non-photochemical energy dissipation of PSII causes a loss in Fo. If reversible deactivation or destruction occurs in the reaction center of PSII, it will increase Fo. Thus, the intrinsic mechanism of this change can be reflected by changes in the initial fluorescence. After being infected by smut, the Fo of the leaves of sugarcane seedlings showed a trend of increasing first and then decreasing with the prolongation of infection time; Fm showed a trend of reducing rather than increasing (Figure 1). The pathogen infection induced the reversible inactivation or destruction of the PSII reaction center for the first day. The Fo of the sugarcane leaves showed a downward trend with the infestation time, indicating that the energy absorbed by the antenna pigment of the PSII of the sugarcane leaves was dissipated in the form of fluorescence and heat. At the same time, the amount of flow to photochemistry is reduced. It can be seen that the pathogen infection caused a certain degree of damage to the photosynthetic apparatus of the sugarcane leaves.

**Figure 2:**
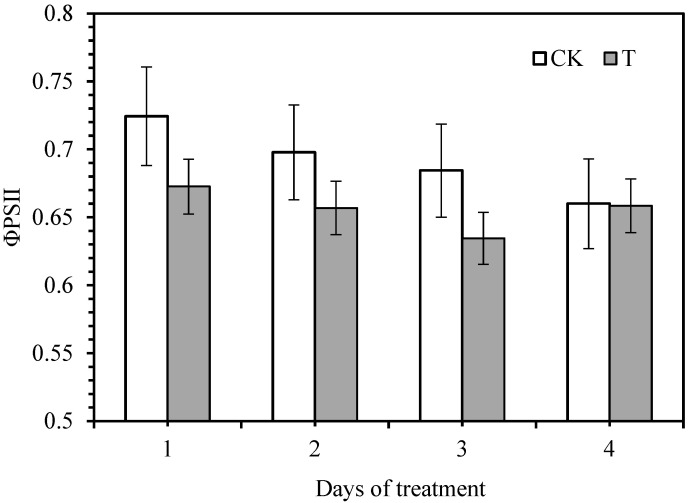
Effect of ΦPSII on sugarcane cv. ROC22 plants during smut inoculation. CK—control, T—smut inoculation.

The maximum photochemical quantum yield (Fv/Fm) reflects the conversion efficiency of the original light energy in the PSII reaction center. It is minimal in plant leaves under non-infected conditions, nearly 0.8. It can be seen from Figure 1 that the original light energy conversion efficiency Fv/Fm shows a trend of decreasing first and then increasing gradually with the treatment time. The difference between Fv/Fm and the control sugarcane seedling leaves reached a significant level, indicating that sugarcane was severely affected 3–4 days after smut inoculation. The actual photochemical efficiency (ΦPSII) is the effective quantum yield of PSII and a relative indicator of the rate of photosynthetic electron transport in plants. This indicator can be directly measured under light conditions without a dark-adapted leaf. It can capture the actual primary light energy of PSII when part of the reaction center is closed. It can be seen from the change of PSII actual photochemical efficiency (ΦPSII), with the prolongation of infestation time, that ΦPSII initially decreased then increased (Figure 2).

**Figure 3:**
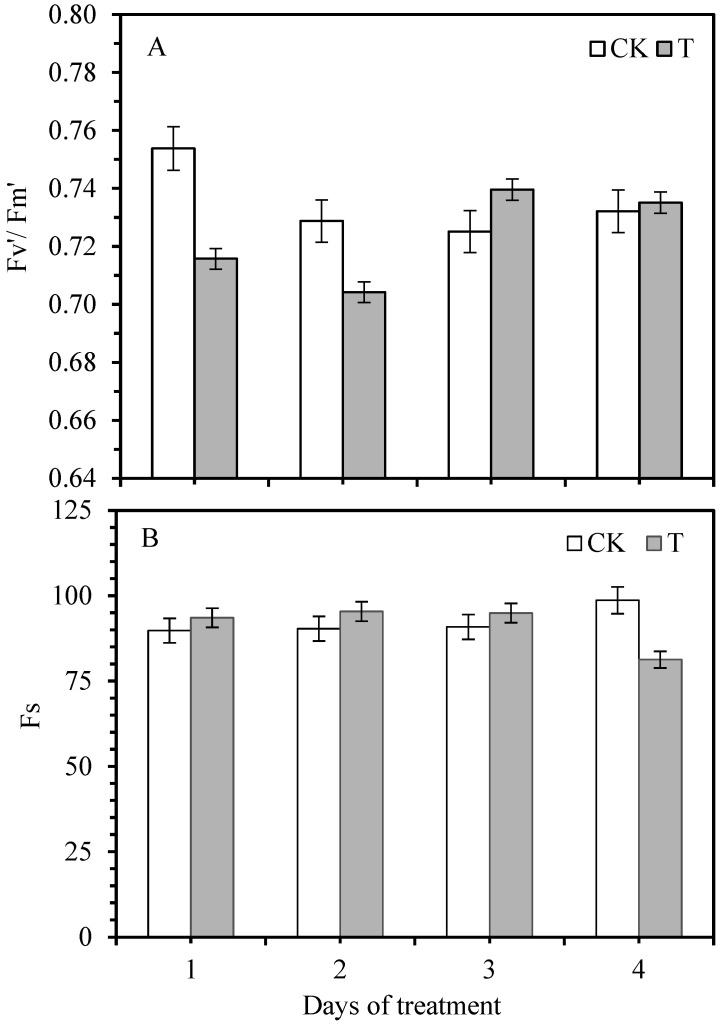
The changes in maximum light energy conversion efficiency ((**A**), Fv′/Fm′) and steady-state fluorescence ((**B**), Fs) of sugarcane cv. ROC22 plant leaves in response to smut inoculation at different time periods. CK—control, T—smut inoculation.

It can be seen from Figure 3 that the maximum light energy conversion efficiency (Fv′/Fm′) of sugarcane seedlings showed decreasing trend initially and then increased as compared to control plants. The overall performance of Fv′/Fm is an upward trend with the infestation time, and the difference between the treatment and the control reached a significant level. The changing trend of Fv′/Fm′ of the PSII reaction center in sugarcane leaves under light adaptation conditions is consistent with the changing trend of Fv/Fm after dark adaptation. Compared with the control, the steady-state fluorescence (Fs) of the light showed an increasing pattern and then decreased; the maximum decrease was observed when pathogen-infected for 4 days. As the pathogen infection time prolonged, the overall trend gradually reduced.

### 3.3. Changes in Photochemical Quenching Coefficient and Non-Photochemical Quenching Coefficient

Fluorescence quenching includes photochemical (qP) and non-photochemical quenching (qNP). Photochemical quenching can reflect the proportion of light energy absorbed by the PSII in photochemical electron transport and indirectly reflect the degree of opening and closing of the PSII reaction center and the ratio of the QA oxidation state. qNP reflects the proportion of light energy absorbed by the PSII in the form of heat, which also demonstrates the energization of the plant photosynthetic membrane. It can be seen from Table 2 that the photochemical quenching coefficient (qP) of the pathogen decreased and then increased as compared to control plants. The difference between the treatment and control reached a significant level, and the increase in qP was the largest with three days of pathogen infection. qNP is opposite to qP, i.e., with the prolongation of infection time, qNP shows a trend of “rise-lower-rise”. Except for the 1-day infestation, the other time treatments were significantly different from the control. The magnitude of qNP reduction was most significant on the third day after being infected by pathogens. This indicates that when sugarcanes were infected by smut, it caused an increase in the proportion of the closed part of the PSII reaction center in the sugarcane leaves and hindered the electron flow in the PSII (oxidation lateral reaction center), which further decreased the quantum yield of electron transport.

### 3.4. Correlation Coefficient between Photosynthesis, Stomatal Conductance, Intercellular CO_2_ Concentration, Transpiration Rate, and Water-Use Efficiency

The correlation coefficients of Pn, gs, Ci, Tr, and WUE are shown in Table 3. The Pn of sugarcane seedlings was positively correlated with gs, Tr, and WUE and negatively correlated with Ci. It can be seen that gs, Tr, and WUE are the main factors affecting Pn.

### 3.5. Correlation Coefficient between Leaf Photosynthetic Parameters and Chlorophyll Fluorescence Variables 

During smut inoculation, the Ci was significantly positively correlated with Fv′/Fm′ and ΦPSII; the initial fluorescence (Fo) positively correlated with Fv/Fm, Fs, and ETR; Fv/Fm positively correlated with Fv′/Fm; Fs significantly positively correlated with Fv′/Fm′; and Fv′/Fm′ positively correlated with ΦPSII. The results showed a close relationship between the photosynthetic responses and chlorophyll fluorescence variables of sugarcane seedlings in response to smut infestation of sugarcane plants (Table 4).

### 3.6. Effects of Smut Infection on Enzymatic Activities in Sugarcane Plants

**Figure 4:**
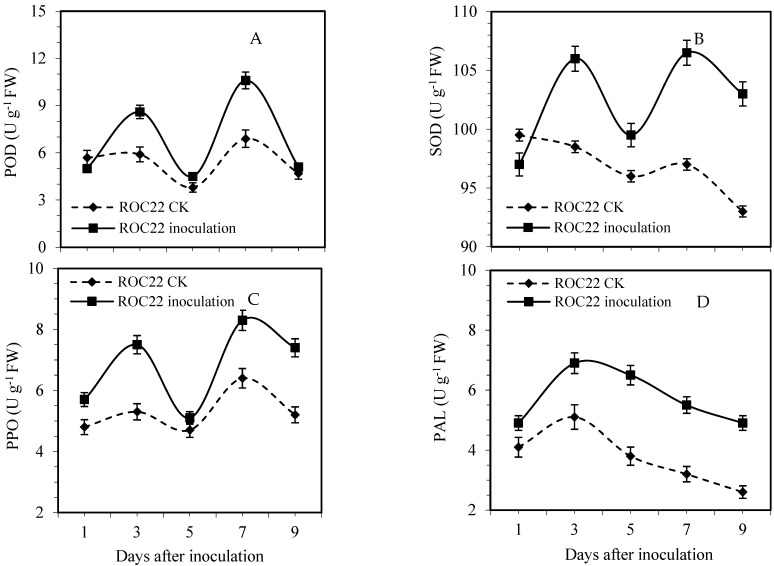
Effect of smut infection on peroxidase ((**A**), POD), superoxide dismutase ((**B**), SOD), polyphenol oxidase ((**C**), PPO), and phenylalanine ammonia-lyase ((**D**), PAL) activities in sugarcane cv. ROC22 plants at specific time intervals.

The results of POD activity determination after inoculation with smut are shown in Figure 4A. The POD activity was increased after inoculation with smut, and the POD activity showed a bimodal trend; the activity reached peaks I and II, respectively. On the third and seventh days after smut inoculation, peak II was more significant than peak I. Compared with the control plants, the POD activity of peak I and II of ROC22 increased by 45.9 and 51.9%, respectively, and the difference reached a significant level (*p* < 0.01).

Figure 4B shows that the SOD activity in sugarcane plant leaves increased after inoculation, and SOD activity peaks were generated or tended to reach the activity peak I and II on the third and seventh days after inoculation, respectively. Compared with the control, ROC22 activity peaks I and II increased by 7.3 and 4.6%, respectively, but the difference was insignificant (*p* < 0.05). It can be seen from Figure 4C, that PPO activity was enhanced after smut inoculation, and reached peak I and peak II activity on the third and seventh days. Peak II was more significant than peak I, respectively. Compared with the control, the activity peak I and II of ROC22 increased by 36.5 and 26.9%, respectively, with a significant difference (*p* < 0.05). As shown in Figure 4D, PAL activity was higher than that of the control during the inoculation period, and increased initially and then decreased, with the peak activity on the third day after inoculation. PAL activity was significantly higher (36.5%) than the control after the third day of smut inoculation.

## 4. Discussion

The influence of sugarcane smut pathogens on sugarcane seedlings is multi-faceted and multi-layered. Green plants synthesize organic matter and gain energy through photosynthesis. Pathogen stress can affect the activity of enzymes related to photosynthetic electron transport and dark reactions in plants, and it can also cause direct damage to the photosynthetic apparatus system. Therefore, the impact of pathogens on plant photosynthesis is multifaceted [5,19]. In this study, the photosynthesis rate of sugarcane seedlings showed a “rising-lowering-liter” change after the artificial stab inoculation of smut pathogens. The photosynthetic rate was observed to have significant reductions on the second and third days after smut infection, and a more significant increase began to occur on the fourth day. Leaf gas exchange in plants between in vivo and in vitro conditions is mainly done through stomata, so changes in gs affect plant Tr and Pn. Plants can regulate the concentration of CO_2_ and the loss of water in plants by changing the opening and closing of their pores or the size of the pores. Therefore, the gs can directly reflect changes in the physiological activity of the plant. The study found that the changes in gs of sugarcane seedlings after smut infection were insignificant. It is considered that there are two main reasons for the decline of Pn; stomatal and non-stomatal factors are mainly affected by the regulation mechanism [2,5,20]. 

It is speculated that the changes in the Pn of sugarcane seedlings are caused by non-stomatal factors during smut infection. In addition, the study also found that the Ci level in sugarcane plants increased after smut infection. This result may be due to the increase in photorespiration of sugarcane seedlings caused by the infection of the pathogen, decreasing the photosynthetic capacity of leaves, which made the supply capacity of CO_2_ exceed the ability of the photosynthetic mechanism to assimilate. In addition, the obstruction of the photosynthetic product transport leads to the accumulation of photosynthetic products in leaves, an important reason for the decrease in Pn. It is speculated that the proliferation of pathogen in the early stage of smut infection inhibits the output of leaf photosynthetic products, which is another possible reason for the decrease in the Pn of sugarcane plants. When the pathogens multiply in the sugarcane plants, they stimulate the excessive Pn of the sugarcane seedlings, eventually leading to excessive nutrient consumption and pre-existing length. Yu et al. [21] found that the trend of Pn and Tr infected with acne scars was consistent with this study. The chlorophyll degradation in leaves is caused by pathogen infection [21].

The change of chlorophyll fluorescence is closely related to the photosynthetic performance. The chlorophyll fluorescence kinetic changes can reflect the effects of stress on the different processes of plant Pn. Therefore, chlorophyll fluorescence parameters can be used to evaluate the function of the plant photosynthetic system and analyze the effects of environmental stress on plants and the extent of damage and the degree of damage in the photosynthetic structure during the adversity. Schnettger et al. [22] suggested that the destruction of the PSII reaction center led to an increase in Fo and a decrease in Fv, Fm, and ΦPSII, which was consistent with the results of the study on sugarcane seedlings 1 and 2 days after smut infection. It can be seen that the infection of smut pathogens caused severe damage to the active center of the PSII in the leaves of sugarcane seedlings, inhibiting the original reaction process and also affecting the photosynthetic electrons from the reaction center of the PSII to the plastids and electron acceptors A and B. In the transmission process, the invasion of pathogens causes photoinhibition in plants. The smut infection causes the decrease in ΦPSII in the sugarcane leaves, indicating that the disease stress reduces the number of electrons involved in CO_2_ fixation and the open ratio of the reaction center in PSII, which leads to the weakening of photosynthetic electron transport ability, the obstruction of the dark reaction of sugarcane leaves, and the slow conversion of light energy captured by photosynthetic pigment into chemical energy [5,23]. 

The ΦPSII and Pn decreases, which will be detrimental to the formation of the final yield of sugarcane. The chlorophyll fluorescence parameters of sugarcane leaves also changed with the duration of pathogen infection. It was found that the smut affected the chlorophyll fluorescence of sugarcane plants. The present findings also found that on the third and fourth day, Fo decreased, and Fv, Fm, and ΦPSII increased after pathogen infection. It can be seen that sugarcane seedlings responded positively by self-regulation in the early stage of smut pathogen infection and reduced their loss.

After smut inoculation, the Fv/Fm value increased initially and then decreased. It is inconsistent with the loss of Fv/Fm value caused by tobacco mosaic virus infection [23]. This may be due to the period of measurement of the initial stage of smut infection, and that sugarcane seedlings have more resistance and regulation to the invasion of the pathogen. The loss of Fv′/Fm′ of sugarcane seedlings on the first and second day after pathogen infection was more significant than that of Fv/Fm, indicating that the rate and efficiency of the original light energy converted into chemical energy were caused by pathogen infection. The energy of light was not as sufficient as that of control, which may be another reason for the decreased photosynthetic rate caused by the smut infection. qP reflects the share of light energy absorbed by the PSII for photochemical electron transport, which also reflects the reduction state of the PSII primary electron acceptor QA. The larger the qP, the more electron transfer activity of the PSII [24]. The qP value of sugarcane seedlings decreased during the first 2 days after smut infestation and reduced power consumption caused by the acyclic electron transfer process was lower, which also indicated that the reduction degree of QA was higher, the proportion of the open part of the PSII reaction center decreased, and the balance of the closed position of the reaction center increased. Stable charge separation cannot be completed when the PSII reaction center is closed. Therefore, it is impossible to realize the ordered linear transfer process of photosynthetic electrons [25]. The qP value increased rapidly on the third and fourth days after infestation, reflecting that the sugarcane seedlings responded positively to the invasion of smut pathogens and reduced their damage.

The activity of POD and SOD in sugarcane was increased after smut infection. Under normal conditions, the reactive oxygen removal system in the plant, such as SOD, POD, etc., keeps the reactive oxygen metabolism in low dynamic equilibrium. Still, the pathogen infects the host and causes the sudden onset of reactive oxygen species (ROS) in the infected parts of the plants. The accumulation of ROS in sugarcane caused by smut led to increased SOD and POD activity which indicated that the sugarcane could reduce the injury to sugarcane by improving SOD and POD activity. PPO is mainly involved in the oxidation of phenols to form strontium and the polymerization of lignin precursors. PAL is an important and rate-limiting enzyme in the phenylpropanoid metabolic pathway. The roles of these two enzymes, mainly PPO and PAL, promote the production of phenolic compounds with antibiotic properties, killing the host’s cells while killing the infected pathogens. PPO and PAL are involved in the synthesis of lignin, while lignin itself is toxic to germs and has an antimicrobial effect. PPO and PAL activities were enhanced after smut infection, indicating involvement in the process of sugarcane response to smut [5,19].

In addition, photoinhibition occurred in the early stage of infection, and photosynthetic capacity increased on the third day, which might be related to increased enzyme activity. Under natural conditions, when plants are subjected to various environmental stresses, the photosynthesis ability of plants is reduced, resulting in inevitably generating excess excitation energy [26]. Plants have developed a series of protective mechanisms during long-term evolution, such as heat dissipation, light respiration, etc., which depend on the xanthophyll cycle. Among them, the Mehler reaction is also considered to play a role in excess light energy dissipation [27,28]. It is speculated that its role may be reflected in two aspects: direct consumption of excess excitation electrons and the establishment of a transmembrane proton gradient to initiate heat dissipation [29]. It showed apparent photoinhibition on the first and second day and recovered on the third day; additionally, the activity of SOD and POD increased, which may be the result of the start of the Mehler reaction. The Mahler reaction is the process by which O_2_ is reduced to O_2_^−^ as an excited single electron acceptor [30]. The O_2_^−^ produced by this reaction converts harmful superoxide radicals into H_2_O_2_ by SOD. Although hydrogen peroxide is still toxic to the body, the body’s CAT and POD immediately break it down into completely harmless water. The three enzymes form a complete antioxidant chain [19,31]. 

Quinone can intervene in the reaction of photosynthesis. PPO only exists in those chloroplasts that produce high-level oxygen and is related to chloroplasts with a high ratio of chlorophyll. While the strong-offset PPO preparation of KCN can significantly improve the release of oxygen in photosynthesis, the broad bean PPO and the PSII protein are co-separated. The first 15 amino acids of the N-terminus of spinach PPO were identical to those of the PSII light-harvesting complex (LHCII) [32]. Therefore, PPO may act as a metal oxidoreductase, regulating the redox level in the cytoplasm, binding to oxygen, and delivering molecular oxygen to regulate the harmful photooxidation reaction rate in the chloroplast, participate in the electron transfer, and function as an energy conversion [33,34].

## 5. Conclusions

This study can help to understand the effects of sugarcane smut pathogens on the photosynthesis, chlorophyll fluorescence, and related defense enzymes of sugarcane seedlings. The Pn, gs, and Tr rates of sugarcane seedlings decreased on the first three days after smut infection and increased on the fourth day. Intercellular CO_2_ concentration increased in the first three days after smut infection and decreased on the fourth day. The chlorophyll fluorescence parameters Fv, Fm, ΦPSII, Fv/Fm, and Fv′/Fm′ decreased at the initial stage of pathogen infection but increased rapidly on the third day after infection. It can be seen that sugarcane seedlings showed a positive response to pathogen infection. Correlation analysis showed that the correlation coefficient between Pn, gs, and Tr reached above 0.800, indicating a significant positive correlation; Ci was significantly positively correlated with Fv′/Fm′ and ΦPSII; Fo positively correlated with Fv/Fm, Fs, and ETR; Fv /Fm significantly positively correlated with Fv′/Fm′; Fs positively correlated with Fv′/Fm′, and Fv′/Fm′ significantly positively correlated with ΦPSII. After inoculation, the related defense enzymes POD, SOD, PPO, and PAL were increased and were associated with the sugarcane response to the smut. The upregulation in photosynthetic capacity may be interconnected to the increase in enzymatic activities.

## Figures and Tables

**Table 1:** The changes in photosynthetic responses, i.e., net photosynthetic rate (Pn), stomatal conductance (gs), intercellular CO_2_ concentration (Ci), rate of transpiration (Tr), and photosynthetic water-use efficiency (WUE) during the inoculation of smut pathogen in sugarcane cv. ROC22.

Photosynthetic Response	Treatment Condition	Days of Smut Inoculation				S	R
		1	2	3	4		
Pn (μmol m^−2^ s^−1^)	CK	14.9 ± 0.72 ^a^	18.9 ± 0.10 ^a^	15.4 ± 0.35 ^a^	19.6 ± 0.62 ^b^	2.331	0.978
	T	15.9 ± 0.74 ^a^	17.7 ± 0.18 ^b^	10.7 ± 1.23 ^b^	25.2 ± 0.92 ^a^	7.233	0.837
gs (mol m^−2^ s^−1^)	CK	0.077 ± 0.005 ^a^	0.133 ± 0.002 ^a^	0.081 ± 0.003 ^a^	0.128 ± 0.010 ^a^	0.031	0.912
	T	0.092 ± 0.008 ^a^	0.127 ± 0.004 ^a^	0.078 ± 0.006 ^a^	0.130 ± 0.029 ^a^	0.030	0.917
Ci (µmol mol^−1^)	CK	54.93 ± 2.93 ^a^	108.33 ± 0.67 ^a^	55.27 ± 8.03 ^b^	100.53 ± 1.85 ^a^	31.063	0.863
	T	72.23 ± 11.15 ^a^	113.33 ± 3.84 ^a^	131.67 ± 7.69 ^a^	97.06 ± 7.55 ^a^	20.155	0.960
Tr (mmol m^−2^ s^−1^)	CK	2.410 ± 0.153 ^a^	3.610 ± 0.063 ^a^	2.523 ± 0.121 ^a^	3.397 ± 0.271 ^a^	0.623	0.952
	T	2.950 ± 1.182 ^a^	3.497 ± 0.029 ^a^	2.367 ± 0.152 ^a^	3.607 ± 0.711 ^a^	0.689	0.944
PWUE (μmol CO_2_ mmol H_2_O^−1^)	CK	6.185 ± 1.021 ^a^	5.230 ± 0.801 ^a^	6.104 ± 0.871 ^a^	5.432 ± 0.832 ^a^	1.601	0.901
	T	5.391 ± 0.924 ^a^	5.147 ± 1.023 ^a^	4.472 ± 0.591 ^a^	5.315 ± 1.201 ^a^	1.623	0.867

Note: CK: inoculation with ddH_2_O, T: inoculation with smut pathogen. S: standard error, R: correlation coefficient. The superscript letters represent a significant difference between different treatments (*p* < 0.05 LSD), *n* = 5.

**Table 2:** The variation of qP and qNP characteristics during smut inoculation.

Fluorescence Parameters	Treatment	Days of Smut Inoculation				Loss or Gain (%)
		1	2	3	4	
qP	CK	0.959 ± 0.003 ^a^	0.949 ± 0.003 ^a^	0.903 ± 0.007 ^b^	0.923 ± 0.008 ^b^	−0.04
	T	0.948 ± 0.004 ^b^	0.930 ± 0.004 ^b^	0.956 ± 0.003 ^a^	0.948 ± 0.002 ^a^	−0.01
qNP	CK	0.253 ± 0.017 ^a^	0.230 ± 0.019 ^b^	0.321 ± 0.021 ^a^	0.137 ± 0.020 ^b^	−0.46
	T	0.245 ± 0.011 ^a^	0.286 ± 0.011 ^a^	0.167 ± 0.009 ^b^	0.278 ± 0.017 ^a^	0.13

Note: CK: inoculation with ddH_2_O, T: inoculation with smut pathogen. Means labeled by different letters are significantly different at *p* < 0.05 using the LSD test.

**Table 3:** The correlation coefficient relationships between photosynthetic parameters.

Variable	Pn	gs	Ci	Tr	WUE
Pn	1.000				
gs	0.926 *	1.000			
Ci	−0.461 **	−0.093	1.000		
Tr	0.984 **	0.978 **	−0.295	1.000	
WUE	0.838 **	0.577 **	−0.864 **	0.727 **	1.000

Note: * and ** indicate significant difference at *p* < 0.05 (r = 0.3291), *p* < 0.01 (r = 0.4238).

**Table 4:** The correlation coefficient of photosynthetic and chlorophyll fluorescence parameters.

Variable	Pn	gs	Ci	Fo	Fv/Fm	Fs	Fv′/Fm′	ΦPSII	qP	qNP	ETR
Pn	1.000										
gs	0.995	1.000									
Ci	0.301	0.283	1.000								
Fo	0.457	0.525	0.554	1.000							
Fv/Fm	−0.484	−0.537	−0.693	−0.984	1.000						
Fs	0.360	0.416	0.714	0.971	−0.990	1.000					
Fv′/Fm′	−0.265	−0.267	−0.978	−0.691	0.804	−0.834	1.000				
ΦPSII	−0.359	−0.345	−0.997	−0.603	0.736	−0.750	0.982	1.000			
qP	−0.757	−0.698	−0.662	−0.181	0.317	−0.241	0.533	0.677	1.000		
qNP	0.062	−0.020	0.706	−0.191	0.012	0.009	−0.554	−0.667	−0.699	1.000	
ETR	0.114	0.211	0.091	0.846	−0.744	0.756	−0.290	−0.137	0.352	−0.634	1.000

*p* < 0.05 (r = 0.3291), *p* < 0.01 (r = 0.4238).

## Data Availability

Not applicable.
